# Variability of Lower Limb Artery Systolic–Diastolic Velocities in Futsal Athletes and Non-Athletes: Evaluation by Arterial Doppler Ultrasound

**DOI:** 10.3390/ijerph17020570

**Published:** 2020-01-16

**Authors:** Pedro Duarte-Mendes, Rui Paulo, Patrícia Coelho, Francisco Rodrigues, Vasco Marques, Sónia Mateus

**Affiliations:** 1 Department of Sports and Well-being, Polytechnic Institute of Castelo Branco, 6000-266 Castelo Branco, Portugal; ruipaulo@ipcb.pt; 2 Sport, Health & Exercise Research Unit (SHERU), Polytechnic Institute of Castelo Branco, 6000-266 Castelo Branco, Portugal; patriciacoelho@ipcb.pt (P.C.); franciscobrodrigues@ipcb.pt (F.R.); 3 Research Unit in Education and Community Intervention (RECI), 3515-776 Viseu, Portugal; 4 Department of Clinical Physiology, Polytechnic Institute of Castelo Branco, 6000-767 Castelo Branco, Portugal; 5 Quality of Life in the Rural World (Q-Rural), Polytechnic Institute of Castelo Branco, 6000-767 Castelo Branco, Portugal; 6 Department Biomedical Sciences Laboratory, Polytechnic Institute of Castelo Branco, 6000-767 Castelo Branco, Portugal; 7 Vascular Ultrasound Laboratory, Angiology and Vascular Surgery Service, University Hospital Centre of Lisbon University Hospital Centre of Lisbon, 1169-024 Lisboa, Portugal; vmarques9@gmail.com; 8 EPE–Neurovascular and Cardíac Ultrasound Lab, Espiríto Santo of Évora Hospital, 7000-811 Évora, Portugal

**Keywords:** arteries, athletes, doppler, lower limb, ultrasonography, velocities

## Abstract

*Background*: Sports athletes, namely high-intensity practitioners, suffer from vascular remodeling overtime. The purpose of this study was to analyze the systolic and diastolic velocities’ variation between non-athletes and futsal athletes by means of arterial lower limb doppler ultrasound. Additionally, we intended to verify if the velocity variations occur primarily at the systolic or the diastolic level and in which arteries. *Methods*: Seventy-six young males (mean ± SD: 24.9 ± 2.8 years old) volunteered to participate in this cross-sectional study and were divided into two groups: a futsal athletes group (*n* = 38; 24 ± 2.78 years) in the central region of Portugal playing on the 2nd national league with the same level of practice (16 ± 2.4 years of practice) and a non-athletes group (*n* = 38: 26 ± 1.8 years) who did not practice sports regularly and were not federated in any sport. All the subjects agreed to participate in the study with the aim of assessing the arterial lower limb through doppler ultrasound (Philips HD7 echograph with linear transducer 7–12 MHz). *Results*: Differences between groups (*p* ≤ 0.05) in the systolic velocity of the left deep femoral artery (*p* = 0.022; *d* = 0.546, small) and in the right superficial femoral artery (*p* = 0.028; *d* = −0.515, small) were found. We also found differences in the diastolic velocity: in the left common femoral artery (*p* = 0.002; *d* = −0.748, moderate), in the right deep femoral artery (*p* = 0.028; *d* = −0.521, small), in the right superficial femoral artery (*p* = 0.026; *d* = −0.522, small), in the right popliteal artery (*p* = 0.002; *d* = −0.763, moderate), and in the left popliteal artery (*p* = 0.007; *d* = −0.655, moderate). Moreover, the athletes’ group presented the highest mean values, with the exception of the systolic velocity of the left deep femoral artery. In intragroup analysis of variance referring to systolic and diastolic velocities in arterial levels in the right and left arteries, differences were found in all analyses (*p* ≤ 0.05). *Conclusions*: We conclude that futsal athletes of our sample go through a process of changes such as increased blood flow velocity in systolic and diastolic cardiac phase in all studied lower limb arteries, showing that the remodeling occurs regardless of vessel radius. Our results reinforce the existence of vascular remodeling that may vary with the sport and its intensity.

## 1. Introduction

The contribution of sport practice is often considered positive for the well-being and health of the human being [1,2]. Studies indicate that frequent practitioners of sports suffer from vascular remodeling over time [2,3,4]. Remodeling occurs differently between the central and peripheral arteries due to their different structural characteristics (i.e., peripheral arteries are more rigid and have less elastin than the central ones) [5,6]. The remodeling occurs more extensively on the different peripheral arteries, specifically in response to the blood flow and tension patterns to which each vessel is exposed during exercise [4,5,6].

During the aerobic and anaerobic physical activity, it is essential to increase muscle oxygen consumption, and this increase depends on the blood flow increase to the musculature and consequent increase in oxygen uptake in muscles [7]. Increased blood flow occurs due to increased cardiac output and flow redistribution towards musculature [2,7,8,9]. Increased capacity to extract oxygen depends on the musculature vascular network and oxidative capacity [2,7,8,9]. In turn, increased cardiac output during dynamic exercise is mainly due to increases in heart rate, but also to an increase in systolic volume at an early stage. Therefore, in the case of sports with mixed physiological demands (aerobic and anaerobic) with more emphasis in the anaerobic component, athletes have higher levels of cardiac output, they have reduced peripheral resistance, good vasoconstriction during exercise, and good post-exercise vasodilation, which may translate into velocity changes and variations in peripheral vessel blood flow in individuals who frequently practice sports [10,11]. 

It has been of considerable interest to understand the changes and the mechanisms responsible for this, as well as to understand the consequences [12]. It is also expected that exercise by muscle contraction and external compression will produce a reduction of the vascular lumen (ischemia) and stimulate a greater vascular adaptation when compared with vessels with smaller reduction. Ischemic muscle contractions occur naturally during exercise, and changes in arterial diameter appear to improve blood flow efficiency but maintain blood flow velocity [13]. The same authors observed reductions in resting blood pressure in the order of 3 to 4 mmHg compared with values in prolonged exercise. However, it was not clear if these reductions were due to changes in arterial diameter. In contrast, the capacity of skeletal muscle blood flow increases with physical exercise practice due to structural vascular remodeling [14]. Although doppler spectrum is often normal, the athletes’ peak systolic velocity may be highly sensitive to stenosis [15]. 

All reported lumen size variations resulting from physical activity with consequent changes in blood flow are due to the fact that small changes in the diameter of a vessel condition create huge variations in its conductance [11,16]. Any change in diameter in increasing direction induces greater velocity in the central bloodstream of the vessel, which is the fastest. Flow changes may also be related to increased or decreased sympathetic stimulation of peripheral blood vessels, where inhibition dilates the vessels, increasing blood flow and hence the velocity; and its stimulation causes vasoconstriction, leading to reduced blood flow. [16,17].

Hence, despite the existence of several studies referring to vascular changes in athletes, there are questions that have not yet been answered in all physical activity modalities and sports, and since all of them have their own characteristics that can translate into different vascular territories and typology, it is important that it is done. Specific studies are needed to better understand the changes in diameter, blood pressure, blood flow velocities, and arterial stiffness, in different anatomical vascular regions and in different sports, clarifying the possible positive or negative consequences that may result from the practice of a particular sport [14,15]. In fact, some studies have verified negative consequences that come from the excessive or incorrect practice of some athletes [14,18]. 

In order to be able to qualitatively and quantitatively evaluate the morphology and dynamics of flows in the main arteries of the lower limbs and any changes that may arise, there are complementary diagnostic tests, such as arterial and venous peripheral vascular doppler ultrasound. The latter is a noninvasive, painless examination that does not imply the use of radiation and which has high applicability and reproducibility [19].

Thus, the aim of this study was to analyze the systolic and diastolic velocities’ variation between non-athletes and futsal athletes by means of arterial lower limb doppler ultrasound. Additionally, we intended to verify if the velocity variations occur primarily at the systolic or diastolic level and in which studied arteries. We expected to see a greater variation in systolic–diastolic velocities in athletes, mostly in systolic velocities and in the common and superficial femoral arteries, which are the largest arteries of the lower limb.

## 2. Materials and Methods 

### 2.1. Participants

An analytical cross-sectional study was conducted on a sample of 76 male subjects aged between 18 and 30 who were divided into two groups: a group of futsal athletes (*n* = 38) (mean age of 24 ± 2.79 years old) and a group of non-athletes (*n* = 38) (mean age of 26 ± 2.35 years). Individuals who practiced futsal three or more times a week playing on the 2nd national league in the central region of Portugal with the same level of practice (16 ± 2.4 years of practice) were considered athletes. The participants of the non-athletes group answered the Portuguese version of the Habitual Physical Activity Questionnaire [20]. Through this questionnaire it was possible to verify that this group of participants practice different sports, for at least 3 years and on a recreational level, including tennis (*n* = 8), football (*n* = 15), basketball (*n* = 4), handball (*n* = 6), and volleyball (*n* = 5). Those who did not practice once a week, or less than three times, and were not federated in any sport were considered non-athletes, consistent with the criteria adopted in previous studies [21]. The sample was selected at two futsal clubs in the central region. In the athletes group, the goalkeepers were excluded. Inclusion criteria were male athletes or non-athletes without muscle–skeletal or neurologic injuries, conditions, or syndromes diagnosed in the past six months who agreed to participate in the study and underwent arterial lower limb doppler ultrasound. All subjects were informed about the purpose of the study and signed an informed consent form. This study was conducted according to the Helsinki Declaration [22], and all procedures were approved by the local Ethics Committee (14/CE-ESALD/2016).

### 2.2. Instruments

All subjects underwent doppler ultrasound through Philips HD7 echograph with linear transducer 7–12 MHz. The echograph is a device that uses the reflection (echo) generated by ultrasound to produce real-time images of human body structures and organs. It also allows the application of other techniques such as doppler, which allows the assessment of the blood vessels flow (arteries or veins). The probe receives the generated echoes, transforming them into signals, which will be interpreted by a computer and then displayed as an image on the monitor [23,24].

Specifically, in this study, arterial lower limb doppler ultrasonography was performed, in which three techniques were applied: echography, pulsed Doppler, and color-coded doppler. According to the anatomical region and arteries characteristics under study, we chose a 7–12 MHz linear probe [23,24].

### 2.3. Procedures

Researchers asked participants some questions about physical activity and the number of times they exercised and recorded the information. The information from the results of the exams was also recorded. This information was obtained through individual questions.

**Figure 1:**
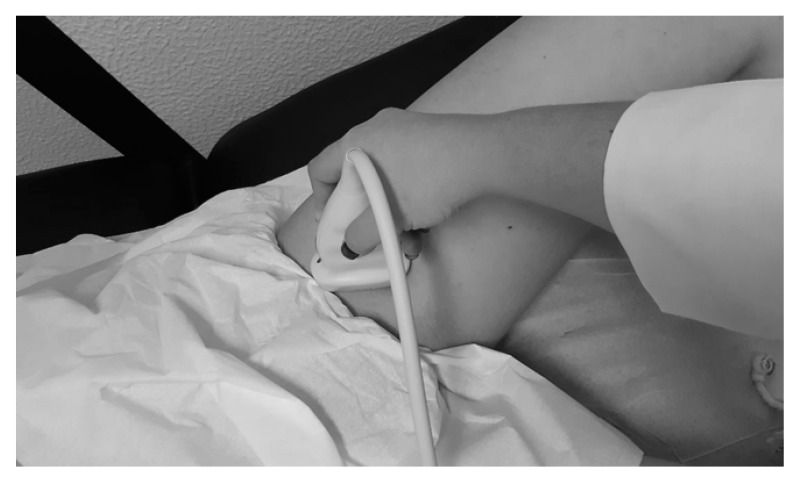
Transducer in position for doppler echo study in the proximal segment of the superficial femoral artery of the left lower limb.

An adequate quiet physical space was required to do the exams, with a low-light environment, a 30 degrees tilt table, an experienced technician, and a 7–12 MHz linear transducer ultrasound. The examinations were performed in the supine position, and three techniques were applied: echography ultrasound, pulsed Doppler, and color-coded doppler (Figure 1).

**Figure 2:**
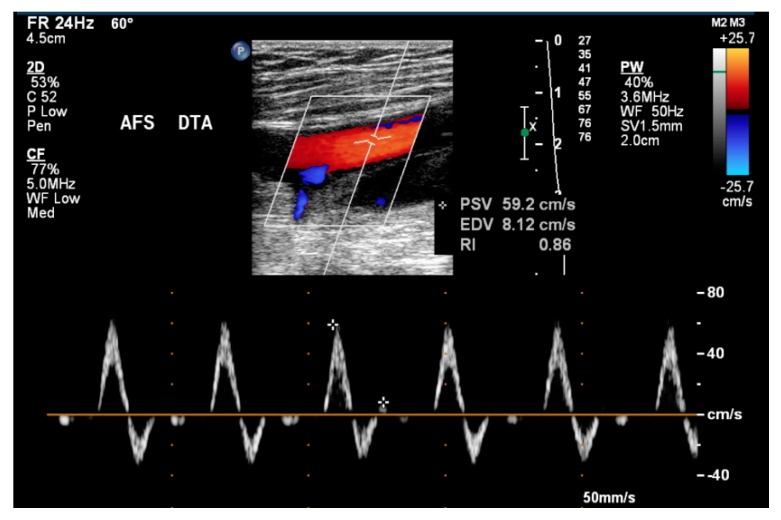
Measurement of systolic and diastolic flow velocities in (cm/s) of the right common femoral artery by lower limb arterial doppler ultrasound.

The ultrasound technique allows for the visualization of the lower limb arterial lumen. Doppler and color-coded doppler techniques were used to verify doppler spectrum morphology and to measure systolic and diastolic flow velocities in centimeters per second (cm/s) about 2 cm after the origin of each artery (Figure 2). Eight arteries were studied in all individuals: common femoral arteries (CFA), superficial femoral arteries (SFA), deep femoral arteries (DFA), and popliteal arteries (PA). The arteries were selected because these are the main arteries suppressing blood flow in the thigh muscles during knee extension exercise, thus being a site of high stress during exercise. All measurements were performed with a fixed 60 degrees angle of insonation and in longitudinal arterial views. The examinations and the results were performed based on normality protocols and criteria [18]. To assist in the performance of the examinations, the National Health Service protocol (2015) on clinical indications and implementation methodology was also used [25]. The same researcher collected all the measurements. Three measurements were taken for each analyzed variable, and the higher value was recorded.

### 2.4. Statistical Analysis

#### 2.4.1. Preliminary Analysis

An inspection of the data revealed no missing values, nor were univariate outliers found. A priori power analysis through G*Power (3.1.9.2) (http://www.psychologie.hhu.de/arbeitsgruppen/allgemeine-psychologie-und-arbeitspsychologie/gpower.html) [26] was used to determine the required sample size considering the following input parameters: effect size *d* = 0.7; *α* = 0.05; statistical power = 0.90. The required sample size was 72 (36 for each group), which was respected in the present study.

#### 2.4.2. Main Statistical Analysis

Descriptive statistics including mean and standard deviation were performed for all variables under analysis. The coefficient of variation (CoV) and the interclass correlation coefficient (ICC) were computed to evaluate the variability of the data. Then, a Shapiro–Wilk test (*n* < 50) to analyze data distribution was performed, considering *p* >0.05 as a normal distribution [27]. Initially, a t-test for independent samples (variables with normal distribution) and Mann–Whitney (variables with non-normal distribution) were used to verify differences between groups (athletes and non-athletes). Secondly, to verify differences across systolic and diastolic velocities in right and left limb on non-athletes and futsal athletes, a Friedman’s test was used, since some of the variables under analysis showed a non-normal distribution, as suggested by several authors [28]. Finally, an effect size (Cohen *d*) analysis was used to determine the magnitude of effect, and the following cut-off values were considered: 0–0.2, trivial; 0.21–0.6, small; 0.61–1.2, moderate; 1.21–2.0, big; >2.0, very big [28]. All statistical analysis was performed using SPSS software v. 25.0 (IBM, Chicago, Illinois, IL, USA), and the significance level was set at *p* ≤ 0.05 to reject null hypothesis [27,29].

## 3. Results

Table 1 shows the differences in the studied variables between the two groups (athletes and non-athletes) on the systolic and diastolic velocities on the right and left arteries. Differences between groups (*p* ≤ 0.05) in the systolic velocity of the left deep femoral artery (*p* = 0.022; small) and in the right superficial femoral artery (*p* = 0.028; small) were found. We also found differences in the diastolic velocity: in the left common femoral artery (*p* = 0.002; moderate), in the right deep femoral artery (*p* = 0.028; small), in the right superficial femoral artery (*p* = 0.026; small), in the right popliteal artery (*p* = 0.002; moderate), and in the left popliteal artery (*p* = 0.007; moderate).

**Figure 3:**
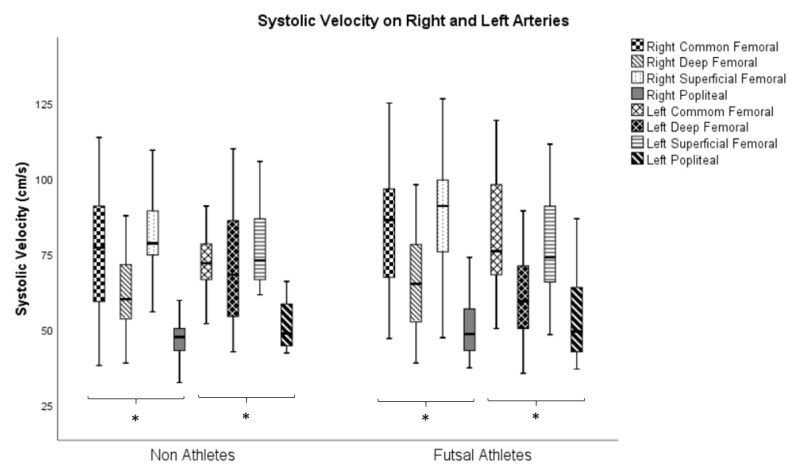
Analysis of the systolic velocities variance on right and left arteries in non-athletes and futsal athletes (* *p* ≤ 0.05—Friedman’s Test).

The analysis of intragroup (athletes and non-athletes) variance referring to systolic velocities in the right and left arteries is present in Figure 3 Differences were found in all the variables under analysis (*p* ≤ 0.05).

With regard to peer comparisons in systolic velocities, on the right side there were differences (*p* ≤ 0.05) in all pairwise comparisons in the studied variables in both groups for the pair common femoral/superficial femoral artery (Table 2). 

Regarding the left side, there were differences (*p* ≤ 0.05) in both groups in the pairs common femoral/popliteal and superficial femoral/popliteal arteries comparisons. In the group of athletes, there were differences in the comparisons of the pairs common femoral/deep femoral and deep femoral/superficial femoral arteries. However, in the non-athlete group, differences were found only in the comparison of the pair deep femoral/popliteal artery.

**Figure 4:**
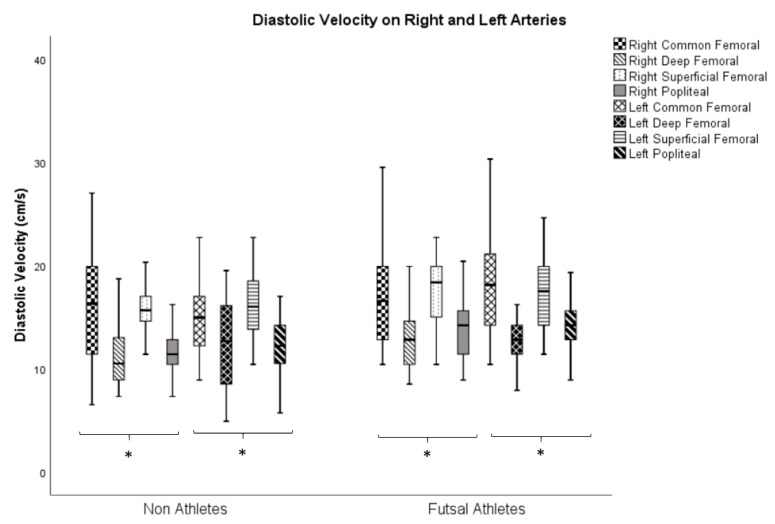
Analysis of the variance of diastolic velocities on right and left arteries in non-athletes and futsal athletes (* *p* ≤ 0.05—Friedman’s Test).

Figure 4 shows the variance analyses related to the diastolic velocities in the right and left arteries. Statistically acceptable values (*p* ≤ 0.05) were found in all analyses for both groups (athletes and non-athletes).

With regard to peer comparisons in diastolic velocities, taking into account the side (right and left) and group (athletes and non-athletes), for the right side there were differences (*p* ≤ 0.05) in all pairwise comparisons in the study variables in both groups, except for the pairs common femoral/superficial femoral and deep femoral/popliteal arteries on both groups (Table 3).

Regarding the left side, there were differences (*p* ≤ 0.05) in both groups in the comparisons of the pairs common femoral/popliteal, deep femoral/superficial femoral, and superficial femoral/popliteal arteries. Additionally, in the athlete group, differences were found in the comparisons of the pairs common femoral/deep femoral and deep femoral/popliteal arteries.

## 4. Discussion

The main purpose of the current study was to analyze the systolic and diastolic velocities’ variation between non-athletes and futsal athletes by means of arterial lower limb doppler ultrasound. Additionally, we intended to verify if the velocity variations occur primarily at the systolic or diastolic level and in which studied arteries. To achieve this, the doppler ultrasonography was chosen as a tool to evaluate arterial velocity changes. This method, similar to in other studies [30,31], was selected because it has advantages such as ease of usage, where measurements can be repeated several times without inconvenience to the individual, as well as being a noninvasive exam [19,32]. Some previous studies aimed to evaluate arterial adaptations in diameter, mean blood flow velocity, and endothelial function of some arteries [2,12]; however, no studies were found where velocity changes were observed in lower limb arteries in futsal athletes. 

Regarding the first objective concerning the analysis of lower limb arteries systolic and diastolic velocities variation between a group of non-athletes and another of futsal athletes through arterial doppler ultrasound, regardless of which limb and phase of the cardiac cycle (systole and diastole), in general statistically significant differences were observed in all the studied arteries (common femoral artery, superficial femoral artery, deep femoral artery, and popliteal artery). The studied arteries in the larger lower limb, the common femoral and the superficial femoral, presented significance in systole, the deep femoral arteries also and the popliteal artery showed significant variation in diastole. These results are in line with previous research [16,17] that states that during exercise there is a need to adapt flow to the increased metabolic activity and oxygen decrease. The cardiovascular system has a function of maintaining homeostatic balance, instigating several changes through regulatory systems, which allows for variable distribution of blood, regulation of blood pressure, and regulation of extrinsic and/or intrinsic factors of muscle activity, notably the vascular radius [16,17]. Anaerobic and aerobic physical activities increase the number of heart contractions per minute, pumping more blood to the arterial vascular system, increasing cardiac output and volume with consequent vasodilatation and increased blood velocity to the straining muscle area to meet local metabolic needs [2].

Another study [3] reported that increased blood pressure and associated hemodynamic during exercise sessions can translate into vascular adaptation, even in distant vascular beds of the active muscle. The same author reported that the physiological adaptations to exercise may depend on the exercise intensity, as well as its modality, duration, and frequency. This, in turn, corroborates with another study that showed that acute exercise induces immediate changes in arterial function, and that exercise perpetuation leads to chronic functional adaptation and, ultimately, structural arterial remodeling [33].

In this study, all futsal athletes had higher average velocity values, except for the left deep femoral artery, where the non-athletes presented the highest average values. That difference can be due to the fact that the deep femoral artery is a secondary artery (branch of the common femoral art) and occasionally, in some individuals, the change in diameter, increase in blood volume and velocity are not very significant. Variations in the velocities found in non-athletes’ femoral arteries may be related to genetic and anatomic issues or by the fact that they are young individuals, despite not exercising more than three times a week.

For the second objective, which was to verify if the variations occur primarily at the systolic or diastolic level and in which studied arteries, by comparing the velocities variation between the different arteries, in both phases of the cardiac cycle, a great number of significant variations in systolic velocities was obtained. However, the difference with diastolic was not very high. In systole, the common femoral arteries’ velocities compared with the superficial femoral arteries’ velocities in athletes and non-athletes were not significant in both limbs, which can be explained by the fact that they are arteries of similar vascular radius, being the difference of the variation of speeds that occurs at each very analogous. Similarly, when comparing diastolic velocities, arteries of a similar radius did not reveal significant differences in velocity variations. In diastole, in addition to the common femoral arteries with superficial femoral and the deep femoral arteries and popliteal arteries of both groups, it was also observed no significant differences. Therefore, in general, the comparison between arteries of different radius showed the most statistical significance regarding the variation of velocities between them, being the artery that showed greater variation in systole was the popliteal artery, whereas in diastole, the superficial femoral and popliteal arteries showed greater variation. Knowing that exercise leads to an increased artery radius, results can be explained by physical laws of conductance, where small changes in the diameter of a vessel condition huge variations in its conductance [11,16]. The ability to conduct laminar blood flow from a vessel increases proportionally to the fourth power of the diameter, and this effect is more evident in the smaller vessel diameter because, in these cases, almost all blood flows very close to the wall, making it very slow [16,17]. Any change in diameter in increasing direction makes a faster velocity in the central bloodstream of the vessel that is the fastest [16,17]. Flow changes may also be explained by the increased or decreased sympathetic stimulation of peripheral blood vessels. While inhibition of sympathetic stimulation dilates vessels, increases blood flow and therefore the velocity, sympathetic stimulation can cause vasoconstriction so that blood flow is reduced [16,17]. Thus, any variation in diameter on small vessels will more easily affect the diastolic velocity, as it is the most sensitive to increases in resistance to blood flow through smaller vessels, and in contrast, large vessel variations reflect more variation in systolic velocities by increasing the velocity of blood flow [16,17]. 

The data presented in the current study reveals certain practical applications. Sports and health professionals should reinforce the importance of maintaining or improving physical exercise or sports with mixed physiological demands (aerobic and anaerobic). That reinforcement can be achieved by recommending regular physical activity practice and sports activities among youth and adults to promote changes in the arterial wall that may contribute to cardioprotective effects of exercise training [34]. Although the present study contributes to knowing how the sports practice influences the systolic and diastolic velocities, it has some limitations. The first being the fact that objective measures were not used to directly assess one or more dimensions of physical activity (e.g., frequency, intensity, time, type) [35], and all variables were assessed at one moment (cross-sectional design). Therefore, we cannot draw causality associations. Other limitations were the comparison of one single sport modality (futsal) against multiple heterogeneous sport activities and the fact that the cofound variables were not analyzed (e.g., hydration, diet, previous physical activities, etc…). Longitudinal and/or experimental studies are needed to further examine the effects of the analyzed variables. In order to increase knowledge on the variability of lower limb artery systolic–diastolic velocities in athletes, we suggest future studies considering the wide range of sport modalities and physical activity. 

## 5. Conclusions

We conclude that in our sample, the futsal athletes had an increase in blood flow velocity in almost all arteries observed in systolic and diastolic cardiac phases, with significant variations in the biggest and smaller arteries, surprisingly showing that the remodeling occurs in all lower limb arteries. These results reinforce the remodeling vascular importance, and we hope that the present study raises awareness among professionals and athletes about the functionality and vascular adaptations that sports with mixed physiological demands (aerobic and anaerobic) can bring. 

Thus, it is important that health and physical activity professionals reinforce the practice of physical activity and sport to promote these vascular alterations in the arterial wall that may have implications in the cardioprotective effect.

Moreover, we should be aware that further research should be developed in this scientific domain and in different sports to estimate, from the medical point of view, the effects of exercise-induced arterial adaptations at different performance levels.

## Figures and Tables

**Table 1:** Descriptive statistics, differences between groups, and effect size in variation of systolic and diastolic velocities.

Variables	Groups	N	M	SD	*p*	*η2*	Effect Size	90% CI	ICC (IC 95%)	CoV
systolic velocity—right common femoral artery	futsal athletes	38	84.86	21.68	0.089 ^b^		−0.396	−0.935–0.143	1 (0.999–1)	0.1%
	non-athletes	38	76.35	21.33			Small		0.963 (0.938–0.980)	1.7%
systolic velocity—left common femoral artery	futsal athletes	38	83.27	22.20	0.068 ^a^	0.044	−0.428		1 (0.999–1)	0.1%
	non-athletes	38	73.99	17.88			Small		0.974 (0.957–0.986)	1%
systolic velocity—right deep femoral artery	futsal athletes	38	66.23	15.12	0.274 ^b^		−0.253	−0.789–0.283	1 (0.999–1)	0.1%
	non-athletes	38	62.68	12.87			Small		1 (0.999–1)	0.1%
systolic velocity—left deep femoral artery	futsal athletes	38	60.61	13.88	0.022 ^a,^*	0.069	0.546		1 (0.999–1)	0.2%
	non-athletes	38	72.14	20.75			Small		1 (0.999–1)	0.2%
systolic velocity—right superficial femoral artery	futsal athletes	38	90.13	19.63	0.028 ^b,^*		−0.515	−1.057–0.028	1 (0.999–1)	0.1%
	non-athletes	38	81.43	13.63			Small		1 (0.999–1)	0.1%
systolic velocity—left superficial femoral artery	futsal athletes	38	77.78	15.73	0.693 ^a^	0.002	−0.091		1 (0.999–1)	0.1%
	non-athletes	38	76.38	12.13			Trivial		1 (0.999–1)	0.1%
systolic velocity—right popliteal artery	futsal athletes	38	51.39	11.00	0.257 ^a^	0.017	−0.262		1(0.999–1)	0.2%
	non-athletes	38	47.38	10.33			Small		1 (0.999–1)	0.2%
systolic velocity—left popliteal artery	futsal athletes	38	54.45	14.08	0.975 ^a^	0.000	−0.007		1 (0.999–1)	0.1%
	non-athletes	38	53.04	11.09			Trivial		1 (0.999–1)	0.1%
diastolic velocity—right common femoral artery	futsal athletes	38	17.18	5.17	0.533 ^a^	0.005	−0.143		1 (0.999–1)	0.9%
	non-athletes	38	16.03	5.90			Tivial		0.996 (0.993–0.998)	0.7%
diastolic velocity—left common femoral artery	futsal athletes	38	18.56	5.09	0.002 ^a,^*	0.123	−0.748		1 (0.999–1)	0.6%
	non-athletes	38	15.19	4.00			Moderate		0.999 (0.998–0.999)	0.9%
diastolic velocity—right deep femoral artery	futsal athletes	38	12.95	2.94	0.028 ^a,^*	0.064	−0.521		0.895 (0.828–0.940)	2.5%
	non-athletes	38	11.55	3.77			Small		0.923 (0.898–0.972)	1.1%
diastolic velocity—left deep femoral artery	futsal athletes	38	12.73	2.33	0.753 ^b^		−0.073	−0.607–0.46	0.972 (0.953–0.985)	1.1%
	non-athletes	38	12.49	3.99			Trivial		1 (0.999–1)	0.8%
diastolic velocity—right superficial femoral artery	futsal athletes	38	17.67	3.31	0.026 ^b,^*		−0.522	−1.065–0.021	0.993 (0.988–0.996)	1.1%
	non-athletes	38	15.95	3.28			Small		0.998 (0.997–0.999)	0.8%
diastolic velocity—left superficial femoral artery	futsal athletes	38	17.57	3.82	0.106 ^a^	0.034	−0.377		1 (0.999–1)	0.5%
	non-athletes	38	16.35	3.93			Small		1 (0.999–1)	0.6%
diastolic velocity—right popliteal artery	futsal athletes	38	13.66	2.74	0.002 ^a,^*	0.127	−0.763		0.999 (0.998–0.999)	0.7%
	non-athletes	38	11.56	2.35			Moderate		0.999 (0.998–0.999)	0.8%
diastolic velocity—left popliteal artery	futsal athletes	38	14.25	2.87	0.007 ^a,^*	0.097	−0.655		0.999 (0.999–1)	0.7%
	non-athletes	38	12.53	3.29			Moderate		0.999 (0.999–1)	0.9%

* *p* ≤ 0.05—^a^ Mann-Whitney U test and ^b^ student’s t-test significance level; *p* > 0.05—Levene’s test equal variances assumed. Note: *N*, subjects’ number; SD, standard deviation; CI, confidence interval; velocity—cm/s; ICC, intra-class correlation coefficient; CoV, coefficient of variation.

**Table 2:** Pairwise comparisons in systolic velocities between arteries in right and left limbs on non-athletes and futsal athletes.

Systolic velocity	Limb	Group	*N*	*p*
common femoral/deep femoral	Right	futsal athletes	38	0.001 *
		non-athletes	38	0.001 *
common femoral/superficial femoral		futsal athletes	38	0.183
		non-athletes	38	0.155
common femoral/popliteal		futsal athletes	38	0.000 *
		non-athletes	38	0.000 *
deep femoral/superficial femoral		futsal athletes	38	0.000 *
		non-athletes	38	0.000 *
deep femoral/popliteal		futsal athletes	38	0.001 *
		non-athletes	38	0.003 *
superficial femoral/popliteal		futsal athletes	38	0.000 *
		non-athletes	38	0.000 *
common femoral/deep femoral	Left	futsal athletes	38	0.000 *
		non-athletes	38	0.286
common femoral/superficial femoral		futsal athletes	38	0.564
		non-athletes	38	0.790
common femoral/popliteal		futsal athletes	38	0.000 *
		non-athletes	38	0.000 *
deep femoral/superficial femoral		futsal athletes	38	0.000 *
		non-athletes	38	0.183
deep femoral/popliteal		futsal athletes	38	0.120
		non-athletes	38	0.000 *
superficial femoral/popliteal		futsal athletes	38	0.000 *
		non-athletes	38	0.000 *

* *p* < 0.05—significance level. Note: *N*, subjects’ number.

**Table 3:** Pairwise comparisons in diastolic velocities between arteries in the right and left limbs on non-athletes and futsal athletes.

Diastolic Velocity	Limb	Group	*N*	*p*
common femoral/deep femoral	right	futsal athletes	38	0.000 *
		non-athletes	38	0.000 *
common femoral/superficial femoral		futsal athletes	38	0.450
		non-athletes	38	0.286
common femoral/popliteal		futsal athletes	38	0.000 *
		non-athletes	38	0.000 *
deep femoral/superficial femoral		futsal athletes	38	0.000 *
		non-athletes	38	0.000 *
deep femoral/popliteal		futsal athletes	38	0.689
		non-athletes	38	0.859
superficial femoral/popliteal		futsal athletes	38	0.000 *
		non-athletes	38	0.000 *
common femoral/deep femoral	left	futsal athletes	38	0.000 *
		non-athletes	38	0.190
common femoral/superficial femoral		futsal athletes	38	0.756
		non-athletes	38	0.076
common femoral/popliteal		futsal athletes	38	0.000 *
		non-athletes	38	0.005 *
deep femoral/superficial femoral		futsal athletes	38	0.000 *
		non-athletes	38	0.000 *
deep femoral/popliteal		futsal athletes	38	0.046 *
		non-athletes	38	0.444
superficial femoral/popliteal		futsal athletes	38	0.000 *
		non-athletes	38	0.000 *

* *p* ≤ 0.05—significance level. Note: *N*, subjects’ number.
